# Chirality Distributions for Semiconducting Single-Walled Carbon Nanotubes Determined by Photoluminescence Spectroscopy

**DOI:** 10.3390/nano11092309

**Published:** 2021-09-06

**Authors:** Masaru Irita, Takahiro Yamamoto, Yoshikazu Homma

**Affiliations:** Department of Physics, Tokyo University of Science, Shinjuku, Tokyo 162-8601, Japan; takahiro@rs.tus.ac.jp (T.Y.); homma@rs.tus.ac.jp (Y.H.)

**Keywords:** carbon nanotubes, photoluminescence, chemical vapor deposition, synthesis

## Abstract

To realize single-walled carbon nanotube (SWCNT) chiral selective growth, elucidating the mechanism of SWCNT chirality (n,m) selectivity is important. For this purpose, an accurate evaluation method for evaluating the chirality distribution of grown SWCNTs without post-growth processing or liquid-dispersion of SWCNTs is indispensable. Here, we used photoluminescence spectroscopy to directly measure the chirality distributions of individual semiconducting SWCNTs suspended on a pillar-patterned substrate. The number of chirality-assigned SWCNTs was up to 332 and 17 chirality types with the chiral angles ranging from $0^{{}^{\circ}}$ to $28.05^{{}^{\circ}}$ were detected. The growth yield of SWCNTs was confirmed to primarily depends on the chiral angle in accordance with the screw dislocation model. Furthermore, when higher-yield chiralities are selected, the chiral angle distribution with a peak corresponding to near-armchair SWCNTs is well fitted with a model that incorporates the thermodynamic effect at the SWCNT-catalyst interface into the kink growth-based kinetic model. Our quantitative and statistical data provide new insights into SWCNT growth mechanism as well as experimental confirmation of theoretical predictions.

## 1. Introduction

Single-walled carbon nanotubes (SWCNTs) [1] are nanoscale tubular materials that exhibit excellent electrical and optical properties because of their pseudo-one-dimensional electronic states [2]. Their growth mechanism is interesting: they grow in a one-dimensional axial direction in the presence of nanoparticles “catalysts” [2]. Because understanding the growth mechanism of SWCNTs is critical for controlling their chirality, which in turn affects their characteristics, many researchers are actively exploring this topic. The growth of SWCNTs with a specific chirality has been achieved using alloy catalysts with high melting points [3,4], and the growth of multi-walled nanotubes several tens of centimeters long have been reported [5]. However, chirality control of SWCNTs has not been established for growth using conventional metal catalysts, such as Fe, Co and Ni, which allow high production efficiency and are important because of their practicality, and growth occurs for only a limited time. The length of SWCNTs prepared using conventional metal catalysts is typically several tens of micrometers. Although SWCNTs were discovered almost 30 years ago, room remains for improving their synthesis method. Further research on the growth mechanisms for SWCNTs is therefore necessary, with the aim of improving growth control.

As a theoretical model of the axial growth of SWCNTs, the kink model, which follows the screw dislocation mechanism in bulk crystals [6], has attracted attention. Ding et al. developed this approach and formulated the chiral-angle dependence of the growth rate [7]. According to this model, the growth rate is approximately proportional to the chiral angle θ. Here, the chiral angle is $0^{{}^{\circ}}$ for a zigzag edge and $30^{{}^{\circ}}$ for an armchair edge. The growth rate increases as the chiral angle increases from zigzag to armchair. In fact, Ding et al. cited existing reports on typical SWCNT synthesis methods, including HiPco [8], CoMoCat [9], alcohol chemical vapor deposition (CVD) [10], and arc discharge [11] methods, and showed that the chiral angle distribution of SWCNTs synthesized using these methods is consistent with this simple model. In addition, Artyuhov et al. considered the thermodynamic effect at the SWCNT-catalyst interface in this kink-growth-based kinetic model and showed that the chiral angle distribution is proportional to $xe^{-x}$ [12], where *x* is defined as x=θ for near-zigzag SWCNTs and $x=30^{{}^{\circ}}-\theta$ for near-armchair SWCNTs. This formula gives a bimodal distribution with peaks corresponding to the near-zigzag and near-armchair SWCNTs; however, because the interface energy is lower for an armchair edge, the model of Artyuhov et al. predicts a chiral angle distribution with a peak corresponding to a near-armchair SWCNT. Artyuhov et al. performed density functional theory calculations for Ni and showed that, if the Ni catalyst is a solid, near-armchair SWCNTs, such as (6,5) and (9,8), are dominant, whereas if the Ni catalyst is a liquid phase, the relative abundance of armchair SWCNTs, such as (6,6) and (9,9), increases.

When experimentally measuring the chirality distribution of grown SWCNTs and comparing the results with theoretical predictions, as previously described, some problems are encountered. Raman spectroscopy [13,14], photoluminescence (PL) spectroscopy [8,15], and transmission electron microscopy (TEM) with electron diffraction (ED) [16,17] are common methods for measuring the chirality of SWCNTs. Raman spectroscopy is used to evaluate the diameter distribution because the radial breathing mode (RBM) frequency of SWCNTs is almost inversely proportional to their diameter [18]. Accurately determining the chirality requires scanning the excitation wavelength and recording the RBM map; however, in general, an edge filter must be used to remove Rayleigh scattering for each excitation laser wavelength. Discrete measurements must be carried out using limited excitation laser wavelengths, and few examples of mapping measurements using numerous laser wavelengths have been reported [19]. In PL spectroscopy, the $E_{22}$ resonant transition, which corresponds to the gap between the second singular states of semiconducting SWCNTs, is generally excited by light in the visible region, and the $E_{11}$ fluorescence emission is observed in the near-infrared region; the chirality can be determined from the combination of the $E_{22}$ and $E_{11}$ energies (wavelengths). However, this method cannot be used for metallic SWCNTs. In addition, when SWCNTs are bundled, energy transfer between the CNTs causes a shift of PL wavelength [20] or even PL quenching, especially if metallic CNTs are present in the bundles. Furthermore, the PL of isolated SWCNTs is also quenched on solid substrates, even on insulators. Consequently, the SWCNTs need to be coated with a surfactant and dispersed in a solution [8], or suspended on a patterned substrate with a pillar-shaped or trench-shaped pattern [15,21]. When SWCNTs are dispersed in a solution with a surfactant, chirality selection likely occurs via a dispersion process. In the case of TEM analysis, with the recent development of aberration correction technology, the honeycomb shape of the tube wall can be observed directly [22]. However, in general, the ED pattern for each SWCNT should be obtained and the acquisition of statistically sufficient data is time consuming. In addition, SWCNTs are susceptible to electron beam damage [23], making TEM a less popular method for evaluating chirality.

As previously described, the existing methods for experimentally evaluating the chirality distribution of SWCNTs are affected not only by the selectivity inherent to the evaluation method itself but also by the chirality or diameter selectivity associated with sample processing (i.e., interaction with a dispersant or the effect of ultracentrifugation). Therefore, few conventional methods provide satisfactory selectivity and statistics. Nevertheless, some studies in which the authors attempted to confirm the screw dislocation model have been reported. Rao et al. used Raman spectroscopy and showed that SWCNTs are likely to grow in proportion to the chiral angle during laser-induced cold-wall CVD [24]; He et al. used TEM to evaluate the SWCNT diameter and chiral angle distributions for different carbon precursors and found that using CO as the carbon source led to a near-armchair preference [25,26].

In the present study, we measured the chirality distribution by PL mapping only for SWCNTs that were singly suspended between micro-pillars grown by CVD. Although the method is applicable to a narrow diameter range and only to semiconducting SWCNTs, it requires neither a dispersant nor a bundle separation process. By devising a pillar-patterned substrate and improving the efficiency of PL mapping measurements, we assigned the chirality of 332 SWCNTs and precisely compared the results with the theoretical predicts on the basis of the highly statistical chiral angle distribution.

## 2. Experimental

### 2.1. Sample Preparation

**Figure 1:**
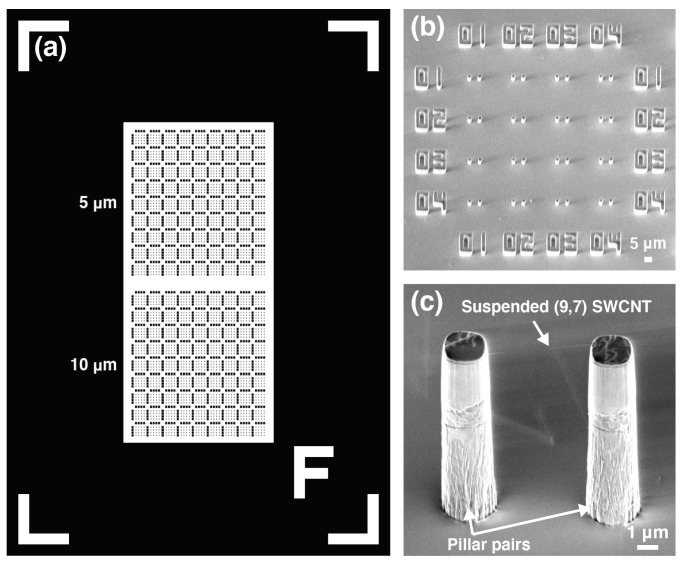
(**a**) Design and (**b**,**c**) SEM images of a pillar-patterned substrate. A chip has 2592 pillar pairs with a height of 10μm and spacings of 5 and 10μm (1296 pairs each).

We used a quartz substrate consisting of pillar pairs manufactured using a lithographic technique [21]. The pillar-patterned substrate contained 2592 pillar pairs with a height of 10μm and spacings of 5 and 10μm, as shown in Figure 1 (1296 pairs each). To synthesize SWCNTs directly on the pillar substrate, a Co catalyst was vacuum deposited onto the pillar substrate to a thickness of 7.8pm. The substrate was introduced into the CVD furnace and heated under an Ar/$H_{2}$ mixed gas (3% $H_{2}$ by volume) at a pressure of $9.3\times 10^{4}\;\mathrm{Pa}$. After the specimens were heated under the Ar/$H_{2}$ mixed gas, ethanol vapor produced by bubbling liquid ethanol with Ar/$H_{2}$ gas was flowed to synthesize SWCNTs. The CVD temperature was varied in the range 750–870 ${}^{{}^{\circ}}$C and the growth time of the SWCNTs was varied in the range 2–14 min. The fabricated SWCNT was shown in Figure 1c. Figure 1c shows a scanning electron microscopy (SEM) image of the fabricated SWCNT between pillar pairs, and the SWCNT chirality was (9,7). We aimed to fabricate a single suspended SWCNT by adjusting the CVD condition to prevent bundling [21].

### 2.2. Methods

#### 2.2.1. PL Measurement System

The synthesized SWCNTs were evaluated using PL measurements under the ambient atmosphere. The PL measurement system comprised a tuneable Ti-sapphire laser (890 Ti:S DW-HN, Coherent, Inc., Santa Clara, CA, USA), an inverted microscope (IX71, OLYMPUS Corp., Shinjuku, Tokyo, Japan) and a grating spectrometer (ACTON Spectra Pro 2300i, Princeton Instruments, Inc., Acton, MA, USA) equipped with an InGaAs one-dimensional array detector (OMA V, Princeton Instruments, Inc., Acton, MA, USA). The excitation wavelength $\lambda_{22}$ for PL spectroscopy was in the range 730–850 nm, and the detection wavelength $\lambda_{11}$ was set to approximately 1000–1500 nm. The laser beam (≃1.6 μm in diameter) was used to irradiate the 2592 pillar pairs on the substrate by moving the sample stage automatically. The positions of synthesized suspended SWCNTs were searched for with $\lambda_{22}=785\;\mathrm{nm}$, and the PL maps were obtained for successfully SWCNT-grown pillar pairs. The PL map was recorded by varying $\lambda_{22}$ from 730nm to 850nm at an interval of 10nm. The spacing between the pillar substrates was designed to be 5 and 10μm in consideration of the laser spot size.

#### 2.2.2. Chirality Assignment

**Figure 2:**
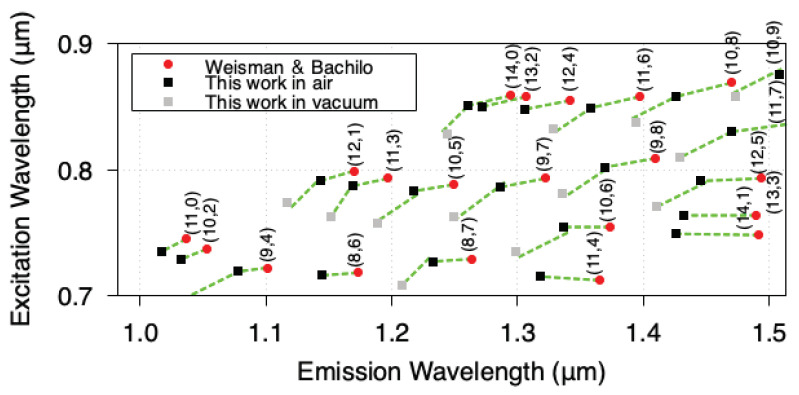
SWCNT excitation and emission wavelengths observable with our experimental setup. The index (n,m) shown in the figure indicates the SWCNT chirality. The red circle is the Weisman–Banchilo empirical data [27], which indicate the experimental results corresponding to an aqueous SDS suspension. The black and grey squares show the present experimental results in air (with water vapor) and in vacuum, respectively.

**Figure 3:**
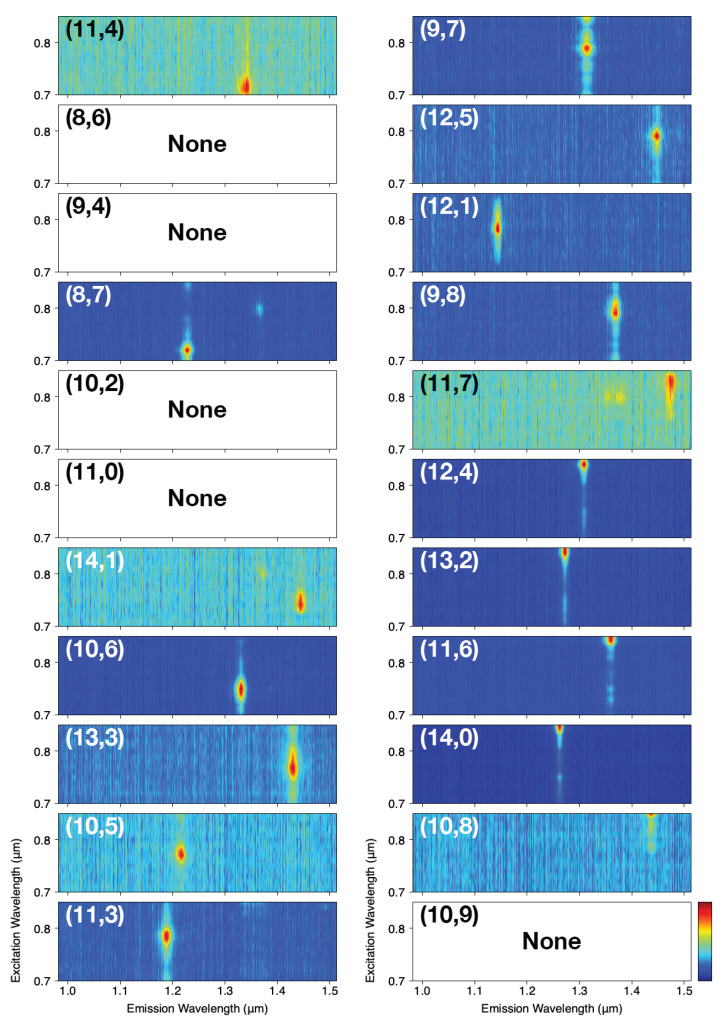
PL maps for suspended SWCNTs for each observed chirality. The index (n,m) in the upper left shows the chirality of the SWCNTs. Chiralities that could not be observed are labelled “None”.

The chirality of a SWCNT can be determined from the combination of its $\lambda_{22}$ and $\lambda_{11}$ values [8]. However, because SWCNTs are a monolayered material, their PL wavelengths are highly sensitive to the surrounding environment. Bachilo et al. first reported the assignment of SWCNT chirality based on a PL map using sodium dodecyl sulfate (SDS)-dispersed HiPco SWCNTs [8]. Weisman and Bachilo later published the empirical data best suited for SDS-wrapped SWCNTs [27]. Several groups have reported environmental effects, i.e., shifts in $\lambda_{22}$ and $\lambda_{11}$ depending on the dielectric environment surrounding SWCNTs [28,29,30]. Miyauchi et al. [31] and Ando [32] published theoretical analyses of environmental dielectric screening on excitons in SWCNTs. However, few data sets have been published for air-suspended SWCNTs [30]. Therefore, we carefully investigated the $\lambda_{22}$ and $\lambda_{11}$ dataset for suspended SWCNTs excited by a Ti-sapphire laser (730–850 nm) in vacuum and in air. In air, water adsorption on the SWCNT surface causes $\lambda_{22}$ and $\lambda_{11}$ to shift from their values in vacuum [33]. Table 1 and Figure 2 compare the detectable chirality in our system with the empirical data for SDS-wrapped SWCNTs reported by Weisman and Bachilo [27]. In our emission/detection wavelength ranges, 22 types of SWCNTs could be detected. The emission/excitation wavelengths from SWCNTs differed depending on the SWCNT chirality, and the plots did not overlap, so each SWCNT chirality could be detected with no error. Notably, weak $\lambda_{22}$ emission was obtained even when the excitation wavelength was off-resonance as evident in the PL maps in Figure 3. Thus, searching only with an excitation wavelength of 785nm was effective for finding all 22 chiralities.

## 3. Results and Discussion

We evaluated the distribution of SWCNT chirality using PL mapping. When two or more semiconducting SWCNTs are bundled and bridged, multiple emission spots are observed in the PL map [20]. In the present study, although we could also observe PL maps from two or more bundled SWCNTs, mainly PL maps from single suspended SWCNTs were observed. Figure 3 shows PL maps from suspended SWCNTs for each chirality. The number of pillars searched was 31,104 (12 substrates) at pillar spacings of 5 and 10μm. The number of SWCNTs assigned a chirality on the basis of the observations was 332 (248 with a pillar spacing of 5μm and 84 with a pillar spacing of 10μm). The percentages of SWCNTs that could be assigned were 1.6% and 0.5% for pillar spacings of 5μm and 10μm, respectively. Thus, the wider pillar spacing rendered the SWCNTs more difficult to suspend. We observed 17 types of SWCNTs among the 22 types listed in Table 1.

**Figure 4:**
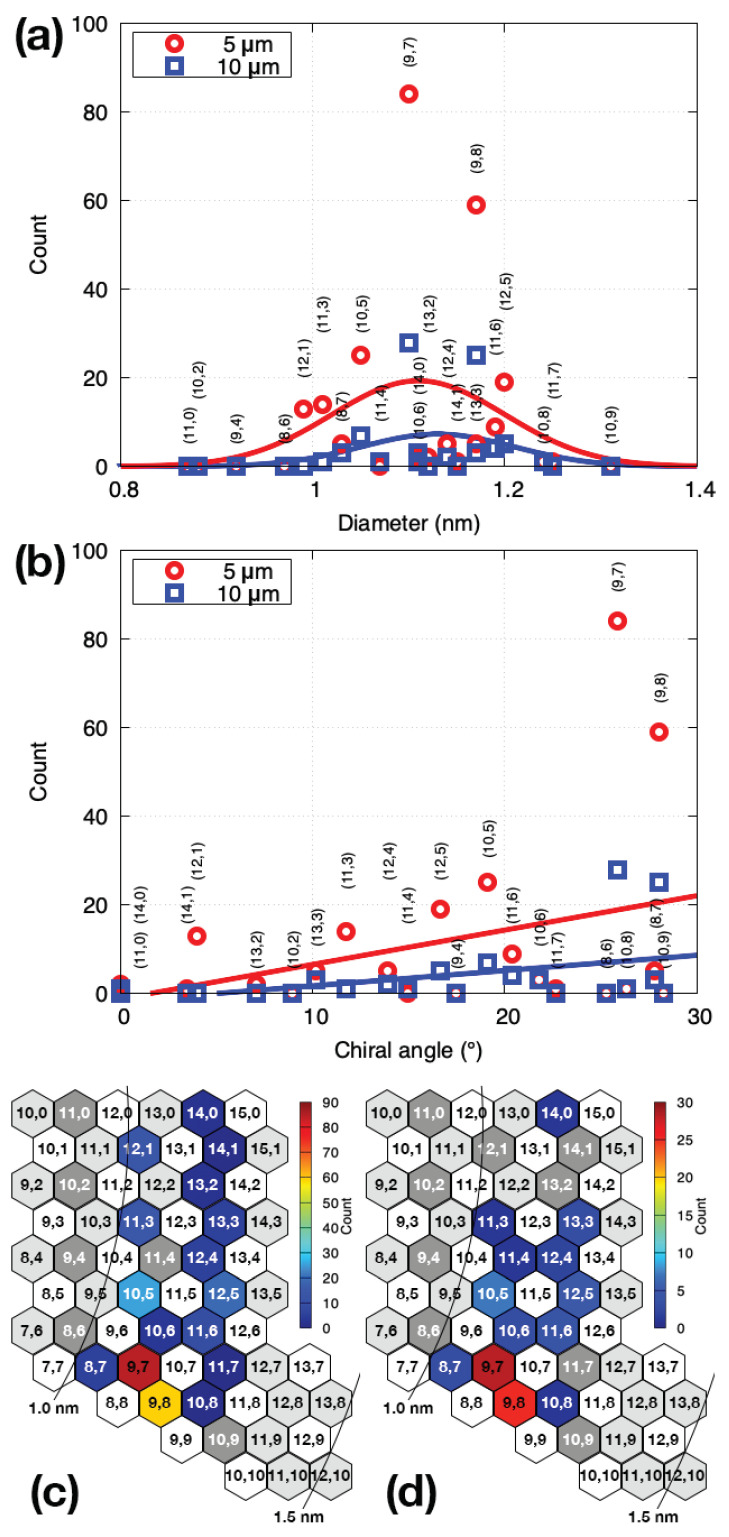
(**a**) Diameter and (**b**) chiral angle distributions for SWCNTs whose chirality could be assigned by PL measurement. The red circles and blue squares indicate the data for pillar spacings of 5μm and 10μm, respectively. Red and blue lines in (**a**) show Gaussian fits for the 5μm spacing (red line: $\mu_{D}=1.11,\;\sigma =0.09$) and 10μm spacing (blue line: $\mu_{D}=1.13,\;\sigma =0.08$). The straight line (aθ+b) fits in (**b**) are for the 5μm (red line: a=0.78,b=1.19) and 10μm (blue line: a=0.34,b=1.72) spacings. (**c**,**d**) Counts and chirality distributions for grown SWCNTs for pillar spacings of 5μm (**c**) and 10μm (**d**). The dark-grey six-membraned rings indicate unobserved SWCNT chiralities, the white ones indicate metallic (or semi-metallic) SWCNTs and the grey ones indicate SWCNTs whose excitation and/or emission wavelengths are out of the measurement range.

Figure 4 shows the diameter and chiral angle tendencies of the grown SWCNTs. The numerical values in the plot are presented in Table A1 in the Appendix A. The most abundant chirality was (9,7): 84 for the 5μm pillar spacing and 28 for the 10μm spacing. The second-most abundance chirality was (9,8): 59 for the 5μm pillar spacing and 25 for the 10μm spacing. Figure 4a shows the diameter distribution of the synthesized SWCNTs. We fitted the data using Gaussian functions and extracted the mean value $\mu_{D}$ and standard deviation σ. In the case of the 5μm pillar spacing, the diameter distribution extended over the range σ=0.09nm centered at $\mu_{D}=1.11\;\mathrm{nm}$. Similar results were obtained for the pillar spacing of 10μm. Figure 4b shows the chiral angle distribution. A straight-line fit was performed under the assumption of a screw dislocation model [7]. The SWCNTs with smaller chiral angles exhibited a lower yield, and the yield was lower for the 10μm pillar spacing than for the 5μm spacing. The formation probability for the suspended structure was lower for the longer spacing [34]: however, the chiral angle dependence was approximately the same for the 5 and 10μm spacings. Therefore, the growth of SWCNTs depended predominantly on the chiral angle. We confirmed that near-armchair SWCNTs were more likely to grow, as predicted by the screw dislocation model [7].

Figure 4c,d show the chirality distributions for the grown SWCNTs on an (n,m) diagram for 5 and 10μm pillar spacings, respectively. From these figures, the diameters and chiral angles of SWCNTs likely to grow can be observed at a glance. They were distributed around (9,7), (9,8) and (10,5) at $\mu_{D}=1.11\;\mathrm{nm}$ and $\theta ≃30^{{}^{\circ}}$. Armchair SWCNTs could not be detected by PL measurements because they were metallic. The only zigzag SWCNTs that could be detected had a chirality of (14,0), with two cases for the pillar spacing of 5μm and one case for the spacing of 10μm. However, the probability was extremely low. We could not find SWCNTs indicated by dark-grey color in Figure 4c,d, which should have been detectable if they existed.

**Figure 5:**
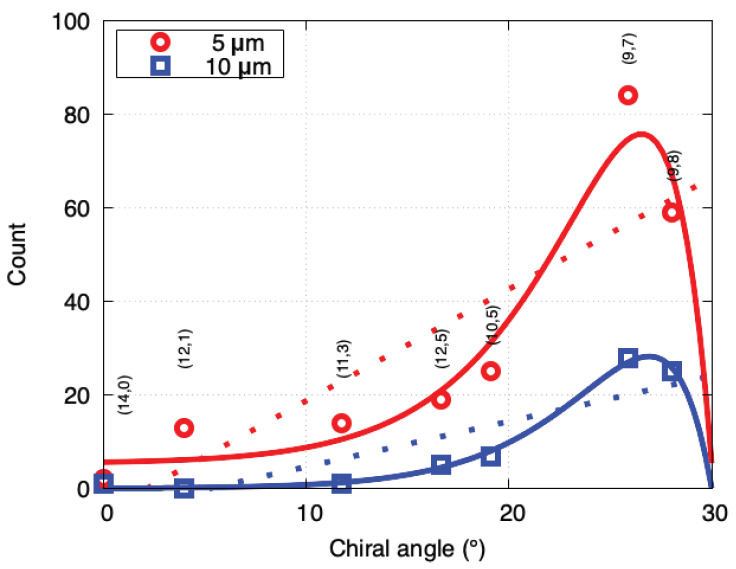
Chiral angle distributions for higher-yield SWCNTs. The straight line (aθ+b) fits are for 5μm (red dotted line: a=2.38,b=5.02) and 10μm (blue dotted line: a=0.97,b=5.04) spacings. The solid curve $a\left(30^{{}^{\circ}}-\theta \right)\exp\left[b\left(30^{{}^{\circ}}-\theta \right)\right]+c$ fits are for 5μm (red line: a=55.29,b=0.29,c=5.37) and 10μm (blue line: a=24.73,b=0.32,c=0.07) spacings.

Notably, the counts of (9,8), (9,7), (10,5), (12,5), (11,3), (12,1) and (14,0) SWCNTs were higher than those of other neighboring chiralities in Figure 4. However, the counts of the (8,7), (10,8), (10,6), (11,6), (11,4) and (12,4) SWCNTs were low, even though the diameter and chiral angle were in the same range as those for the SWCNTs with higher counts. These results indicate that factors other than the chiral angle, such as the cap nucleation probability, also affected the growth probability. We therefore selected the higher-yield SWCNTs and examined their chiral angle distribution (Figure 5). Surprisingly, the experimental data could be well fitted with the model proposed by Artyuhov et al. [12], i.e., $a\left(30^{{}^{\circ}}-\theta \right)\exp\left[b\left(30^{{}^{\circ}}-\theta \right)\right]$, where a and b are fitting parameters. The distribution shape was approximately the same for the 5 and 10μm pillar spacings. The higher yield for (9,7) than for (9,8) was consistent with the model. In the conventional screw dislocation model [7], the chiral angle distribution was proportional to the chiral angle, which failed to explain the experimental results. The model [12] incorporated the thermodynamic effect at the SWCNT-catalyst interface and reproduced the experimental results.

**Figure A1:**
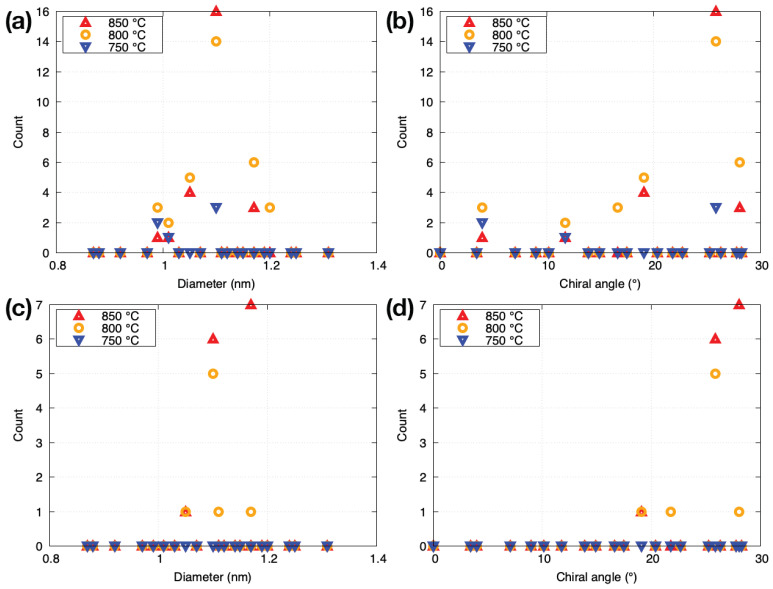
The temperature dependence of the diameter and chiral angle distributions of SWCNT that was able to assign chirality by PL measurement. The pillar spacing is (**a**,**b**) 5μm and (**c**,**d**) 10μm.

**Figure A2:**
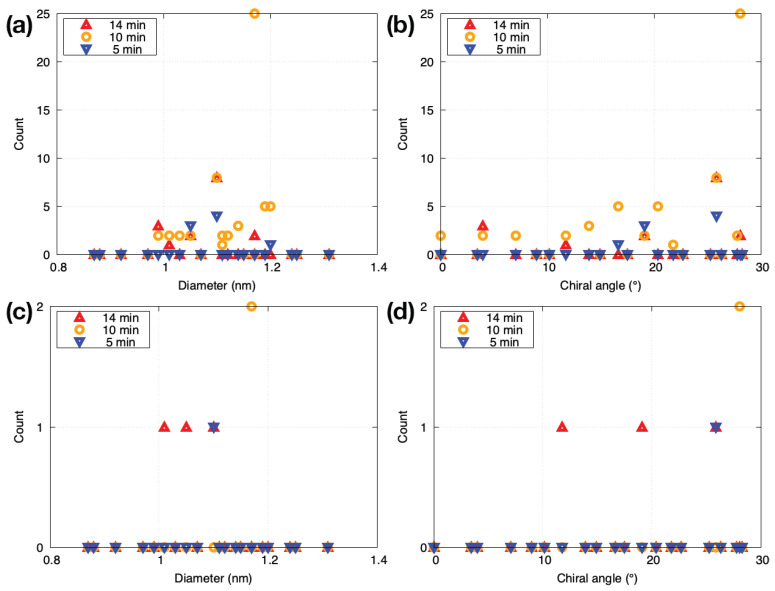
The growth time dependence of the diameter and chiral angle distributions of SWCNT that was able to assign chirality by PL measurement. The pillar spacing is (**a**,**b**) 5μm and (**c**,**d**) 10μm.

The CVD temperature was varied in the range 750–850 ${}^{{}^{\circ}}$C; however, no systematic temperature dependence was observed (Figure A1 in the Appendix A), although the SWCNT yield increased with increasing temperature. We also varied the growth time for the SWCNTs from 5 to 14min; however, we could not observe a systematic relationship with the number of SWCNTs that could be measured by PL (Figure A2 in the Appendix A). In general, a longer growth time produces more SWCNTs. However, when semiconducting SWCNTs are bundled with metallic SWCNTs, PL does not occur; thus a longer growth time does not necessarily increase the number of SWCNTs detectable by PL spectroscopy. The 2–14 min growth time range might be appropriate. The results for different CVD temperatures and growth times in the above ranges are plotted together in Figure 4 and Figure 5.

## 4. Conclusions

We used PL spectroscopy to evaluate the chirality of individual SWCNTs directly synthesized on a pillar substrate without using any dispersant or post-growth processing. The number of detected individual SWCNTs was 332 in total, and 17 chirality types with chiral angles ranging from $0^{{}^{\circ}}$ to $28.05^{{}^{\circ}}$ were assigned. We confirmed that the growth yield of SWCNTs depends on the chiral angle and that near-armchair SWCNTs are dominant, consistent with the predictions of the screw dislocation model. Moreover, SWCNT chiralities with low yields were obtained even though their diameter and chiral angle were in the same range as those for the high-yield SWCNTs. We speculated that factors other than the chiral angle, such as the probability of cap nucleation, also contribute. We would like to continue research and evaluation of other catalysts in the future. Finally, we confirmed that SWCNTs with high yields exhibit a chiral angle distribution with a peak corresponding to near-armchair SWCNTs, in accordance with a model that considers the thermodynamic effect at the SWCNT-catalyst interface in the kink-growth-based kinetic model. The present experimental study provides not only an experimental confirmation of the theoretical predictions but also new insights into controlling the growth of SWCNTs.

## Figures and Tables

**Table 1:** SWCNT chirality (n,m) observable with our experimental setup, along with the diameter *D*, chiral angle θ and excitation and emission wavelengths in each environment. The PL emission/excitation wavelengths from SWCNTs in aqueous SDS suspensions are described as $\lambda_{11}^{SDS}/\lambda_{22}^{SDS}$ [27]. The PL emission/excitation wavelengths for SWCNTs in air (with water vapor) are described as $\lambda_{11}^{air}/\lambda_{22}^{air}$, and those for SWCNTs in vacuum are described as $\lambda_{11}^{vac}/\lambda_{22}^{vac}$.

#	(n,m)	D(nm)	θ(deg)	$\lambda_{11}^{\mathrm{SDS}}\;\left(\mathrm{nm}\right)$	$\lambda_{22}^{\mathrm{SDS}}\;\left(\mathrm{nm}\right)$	$\lambda_{11}^{\mathrm{air}}\;\left(\mathrm{nm}\right)$	$\lambda_{22}^{\mathrm{air}}\;\left(\mathrm{nm}\right)$	$\lambda_{11}^{\mathrm{vac}}\;\left(\mathrm{nm}\right)$	$\lambda_{22}^{\mathrm{vac}}\;\left(\mathrm{nm}\right)$
1	(11,4)	1.07	14.92	1365	712	1318	715		
2	(8,6)	0.97	25.28	1173	718	1145	716	1108	694
3	(9,4)	0.92	17.48	1101	722	1078	719	1043	697
4	(8,7)	1.03	27.80	1264	729	1233	727	1208	708
5	(10,2)	0.88	8.95	1053	737	1033	729		
6	(11,0)	0.87	0.00	1037	745	1017	735		
7	(14,1)	1.15	3.42	1492	748	1426	749		
8	(10,6)	1.11	21.79	1374	754	1337	754	1299	735
9	(13,3)	1.17	10.16	1490	764	1432	764		
10	(10,5)	1.05	19.11	1249	788	1218	783	1189	757
11	(11,3)	1.01	11.74	1197	793	1169	787	1152	763
12	(9,7)	1.10	25.87	1322	793	1286	786	1249	763
13	(12,5)	1.20	16.63	1494	793	1446	791	1411	771
14	(12,1)	0.99	3.96	1170	799	1143	791	1117	774
15	(9,8)	1.17	28.05	1410	809	1370	802	1336	781
16	(11,7)	1.25	22.69	1514	836	1470	830	1429	810
17	(12,4)	1.14	13.90	1342	855	1306	848		
18	(13,2)	1.12	7.05	1307	858	1272	850		
19	(11,6)	1.19	20.36	1397	858	1358	849	1328	832
20	(14,0)	1.11	0.00	1295	859	1261	851	1244	828
21	(10,8)	1.24	26.33	1470	869	1426	858	1394	838
22	(10,9)	1.31	28.26	1555	889	1508	876	1473	858

## Appendix A

**Table A1:** Table of experimental data shown in Figure 3. This table was arranged in the order of chiral angle.

(n,m)	D(nm)	θ(deg)	5μm	10μm
(11,0)	0.87	0.00	0	0
(14,0)	1.11	0.00	2	1
(14,1)	1.15	3.42	1	0
(12,1)	0.99	3.96	13	0
(13,2)	1.12	7.05	2	0
(10,2)	0.88	8.95	0	0
(13,3)	1.17	10.16	5	3
(11,3)	1.01	11.74	14	1
(12,4)	1.14	13.90	5	2
(11,4)	1.07	14.92	0	1
(12,5)	1.20	16.63	19	5
(9,4)	0.92	17.48	0	0
(10,5)	1.05	19.11	25	7
(11,6)	1.19	20.36	9	4
(10,6)	1.11	21.79	3	3
(11,7)	1.25	22.69	1	0
(8,6)	0.97	25.28	0	0
(9,7)	1.10	25.87	84	28
(10,8)	1.24	26.33	1	1
(8,7)	1.03	27.80	5	3
(9,8)	1.17	28.05	59	25
(10,9)	1.31	28.26	0	0
		Parameter	248	84
